# Active Antioxidant Phenolics from Brazilian Red Propolis: An Optimization Study for Their Recovery and Identification by LC-ESI-QTOF-MS/MS

**DOI:** 10.3390/antiox10020297

**Published:** 2021-02-16

**Authors:** Daniel Vieira de Morais, Pedro Luiz Rosalen, Masaharu Ikegaki, Anna Paula de Souza Silva, Adna Prado Massarioli, Severino Matias de Alencar

**Affiliations:** 1 Center for Nuclear Energy in Agriculture, University of São Paulo, Piracicaba, SP 13416-000, Brazil; danielmorais@usp.br; 2 Faculty of Pharmaceutical Sciences, Federal University of Alfenas, Alfenas, MG 37130-001, Brazil; pedro.rosalen@unifal-mg.edu.br (P.L.R.); masaharu.ikegaki@unifal-mg.edu.br (M.I.); 3 Department of Agri-Food Industry, Food and Nutrition, ‘Luiz de Queiroz’ College of Agriculture, University of São Paulo, Piracicaba, SP 13416-000, Brazil; anna.paula.silva@usp.br (A.P.d.S.S.); adnaprado@usp.br (A.P.M.)

**Keywords:** isoflavonoids, bioactive compounds, Box–Behnkendesign, natural products, *Apis mellifera*

## Abstract

Brazilian red propolis (BRP) is a natural product widely known for its phenolic composition and strong antioxidant properties. In this study, we used the Box–Behnken Design (BBD) with Surface Response Methodology to optimize the extraction conditions for total phenolic content (TPC) and Trolox equivalent antioxidant capacity(TEAC) of bioactive phenolics from BRP. The extraction time, ethanol/water concentration and temperature, were tested. All variables had significant effects (*p*  ≤ 0.05), with a desirability coefficient of 0.88. Under optimized conditions (90% ethanol at 80 °C for 30 min), the BRP extract showed a TPC of 129.00 ± 2.16 mg GAE/g and a TEAC of 3471.76 ± 53.86 µmol TE/g. Moreover, FRAP and ORAC assays revealed that the optimized BRP extract had 1472.86 ± 72.37 µmol Fe^2+^/g and 4339.61 ± 114.65 µmol TE/gof dry weight, respectively. Thirty-two phenolic compounds were tentatively identified by LC-QTOF-ESI-MS/MS, of which thirteen were found for the first time in BRP, including four flavones, one flavanol, two flavanones, two chalcones, and four isoflavonoids. Thus, our results highlight the importance of BRP as a source of a wide variety of phenolic compounds with significant antioxidant properties.

## 1. Introduction

Propolis or bee glue is a resinous balsamic substance collected by bees from plant exudates. It is naturally used to protect the hive and as an efficient antiseptic [1,2]. Historically, propolis has been used as a therapeutic substance in folk medicine, but recent advances in science and technology are increasing its commercial value in the food and pharmaceutical industries [3].

Among the different types of propolis occurring worldwide, Brazilian red propolis (BRP) stands out for its health benefits, which are attributed to a phenolic-rich composition, mainly isoflavonoids. The mechanisms of action of some BRP compounds, such as formononetin, vestitol, and neovestivol, were recently examined. These constituents were found to have strong antioxidant, anti-inflammatory, and antimicrobial properties [4,5].

To date, more than 200 compounds have been identified in BRP [4,6,7,8,9], most of which are polyphenols. Propolis composition is causally related to both its botanical source and environmental conditions. The main botanical sources of BRP are *Dalbergia ecastaphyllum*, a rich source of isoflavonoids, and *Symphonia globulifera*, a rich source of polyprenylated benzophenones (guttiferone E and oblongifolin B) and triterpenoids (β-amyrin and glutinol) [9].

Although several studies have correlated the presence of phenolics with the biological activity of BRP, mainly antimicrobial, antioxidant, anti-inflammatory, and anti-cancer properties [10,11,12], an optimization of extraction conditions has not been carried out thus far. The chemical extraction is the initial procedure for recovery of polyphenols from a natural product. Thus, choosing appropriate extraction conditions (e.g., sample-to-solvent ratio, solvent concentration, temperature, and extraction time) is utterly important as these may affect the final extract composition and bioactivity [13].

As stated by Riswanto et al. [14], the Response Surface Methodology (RSM) is a technique widely applied in the optimization of natural products due to its advantages compared to the traditional one-variable-at-a-time design. When combined with an experimental design like the Box–Behnken Design (BBD), it can be employed as a mathematical and statistical tool in natural product research. Due to its capacity of reducing the number of experiments required to find optimal conditions, BBD has been effectively used to optimize polyphenol extraction [13,15].

Several studies have reported the phenolic composition of BRP, but none of them were carried out under optimized extraction conditions. In addition, the few studies addressing atentative identification of phenolicsby High-Resolution Mass Spectrometrywere not carried out in negative mode.Here, the optimization procedureallowedus toidentifyseveral unknownphenolic compounds in the BRP extract.

Our study hypothesis was that the optimized BRP extract had a greater number of phenolic compounds—many of which yet unknown—and stronger antioxidant activity.

Therefore, the aim of this study was to establish the optimal extraction conditions for the recovery of antioxidant compounds from BRP and to evaluate the new phenolic composition of the optimized extracts by LC-ESI-QTOF-MS/MS in negative mode.

## 2. Materials and Methods

### 2.1. Chemicals

The following chemicals were used in this study: Folin–Ciocalteau reagent (DinamicaQuimicaContemporanea, Diadema, SP, Brazil); sodium carbonate, potassium chloride, ethanol (EtOH); monobasic and dibasic potassium phosphate. The standards ({±})-6-hydroxy-2,5,7,8-tetramethylchromane-2-carboxylic acid (Trolox), gallic acid, diammonium salt (ABTS) and potassium peroxydisulfate, fluorescein sodium salt and 2,20-azobis(2-methylpropionamidine) dihydrochloride (AAPH) were purchased from Sigma-Aldrich (St. Louis, MO, USA). All other reagents and solvents were of analytical grade.

### 2.2. Propolis Collection and Extraction

BRP samples were collected from the internal parts of *Apis mellifera* L. (Apidae) beehives located in the city of Maceió (9°40′ S, 35°41′ W), Alagoas State, Northeastern Brazil. Access to the Brazilian genetic heritage was previously obtained in accordance with the Brazilian legislation SECEX/CGEN Ordinance No. 1. Approval for sample collection was obtained via the SISGEN platform under accession number A5A0509.

Propolis samples were crushed with liquid nitrogen, weighted (0.5 g), mixed with 50 mL of solvent and remained in sealed tubes in a bath shaker (Gyromax 929, Amerex) for the time and temperature established in the experimental design (Table 1). After that, BRP ethanolic extracts were kept overnight at −20 °C until complete wax decantation. The supernatant solution was filtered, concentrated on a rotary evaporator at 110 mbar and 50 °C, lyophilized, and then used in the analysis of antioxidant activity and total phenolic content. All extraction procedures were carried out in triplicate.

### 2.3. Experimental Deisgn and Optimization

The following independent variables were considered: Time (X1) (30–90 min), temperature (X2) (30–80 °C), and percentage of ethanol/water (X3) (60–90%, *v*/*v*). Following the Box–Behnken design, 15 experiments with 3 central points were performed to determine the effects of these independent parameters on two dependent responses (TEAC and TPC) (Table 1). RSM was performed to investigate the relationship between the independent and dependent variables. The quadratic polynomial model is represented by the following equation:

(1) $$Y\;=\;b_{0}+\;\overset{3}{\underset{i=1}{\sum }}b_{i}X_{i}+\overset{3}{\underset{i=1}{\sum }}b_{ii}X_{ij}+\overset{3}{\underset{i<1}{\sum }}b_{ij}X_{i}X_{j}$$

where, *Y* is the dependent variable (antioxidant activity) for the independent responses (*X*_1_–*X*_3_); and *β*_0_, *β_i_*, *β_ii_*, and *β_ij_* are constant coefficients of intercept, linear, quadratic, and interaction terms.

### 2.4. Total Phenolic Content

The analysis of total phenolic content (TPC) was performed according to the Folin–Ciocalteau spectrophotometric method, with some modifications. Aliquots of 20 µL of the standard solution (gallic acid) or BRP extract and 100 µL of the Folin–Ciocalteau solution (10% in water) were pipetted into the wells of a microplate. After 5 min, 75 µL of a 7.5% sodium carbonate aqueous solution were added to each well. A control was prepared by replacing the sample with distilled water. The absorbance was measured at 740 nm in a microplate reader (Molecular Devices, LLC, Sunnyvale, CA, USA) after 40 min. The TPC was calculated by linear regression using gallic acid as a standard, and the results were expressed as mg of gallic acid equivalents (GAE) per g of dry extract [16]. All samples were analyzed in triplicate.

### 2.5. Antioxidant Activity

#### 2.5.1. Ferric Reducing Antioxidant Power (FRAP) Assay

Briefly, 20 μL of BRP extract were mixed with 30 mL of water and 200 μL of FRAP reagent (prepared fresh daily) in a 96-well microplate. The FRAP reagent consisted of 10 volumes of 300 mmol/L acetate buffer (pH 3.6), one volume of 20 mmol/L FeCl_3_, and one volume of 10 mmol/L TPTZ in 40 mmol/L HCl [16]. The absorbance was measured at 595 nm in a microplate reader (Molecular Devices, LLC, Sunnyvale, CA, USA) after 8 min. Water was used as a blank; ferrous sulphate solutions (100 to 700 µM) were used for calibration; and the FRAP value was calculated by linear regression. The assay was performed in triplicate, and the results were estimated as µmol Fe^2+^ /g of dry extract.

#### 2.5.2. Peroxyl Radical (ROO^●^)

Briefly, 30 μL of BRP extract plus 60 μL of fluorescein and 110 μL of an AAPH solution were transferred to a microplate. The reaction was performed at 37 °C and the absorbance was measured every minute for 2 h at 485 nm (excitation) and 528 nm (emission) in a microplate reader (Molecular Devices, LLC, Sunnyvale, CA, USA). Trolox standard was used at concentrations ranging from 12.5 to 400 μM. The results were expressed as μmol/Trolox equivalents (TE) per g of dry extract [17]. The assay was carried out in triplicate.

#### 2.5.3. Trolox Equivalent Antioxidant Capacity (TEAC) Assay

The antioxidant capacity of the BRP extract was determined based on free radical ABTS, with modifications [16]. The ABTS radical was diluted in 75 mM potassium phosphate buffer (Ph 7.4) to an absorbance of 0.700 ± 0.01 at 734 nm. Aliquots of 20 µL of Trolox or BRP extract and 220 µL of ABTS radical solution were transferred to the wells and kept at room temperature protected from light. After 6 min of reaction, the absorbance was read at 734 nm using the potassium phosphate buffer as a blank. Trolox was used as a standard at concentrations ranging from 12.5 to 200 µM, and the results were expressed as µmol Trolox equivalents (TE) per g of dry extract.

### 2.6. High-Resolution Mass Spectrometry Analysis (LC-ESI-QTOF-MS/MS)

Liquid chromatography analysis was carried out in a chromatograph (Shimadzu Co., Tokyo) with a LC-30AD quaternary pump and SPD-20A photodiode array detector (PDA). Reversed phase chromatography was performed using a Phenomenex Luna C18 column (4.6 × 250 mm × 5 μM). A high-resolution mass spectrometer (MAXIS 3G—Bruker Daltonics, Bremen, Germany) was equipped with a Z-electrospray (ESI) interface operating in negative ion mode with a nominal resolution of 60,000 *m*/*z*. Twenty microliters of the BRP extract were injected into the liquid chromatography system. The analytical conditions were set as follows: Nebulizer at 2 Bar; dry gas at 8 L/min; temperature at 200 °C and HV at 4500 V. The mobile phase consisted of two solvents: (A) Water/acetic acid (99.5/0.5, *v/v*) and (B) methanol. The flow rate was 1 mL/min, and the gradient was initiated with 30% B, increasing to 40% B (15 min), 50% B (30 min), 60% B (45 min), 75% B (65 min), 75% B (85 min), 90% B (95 min), decreasing to 30% B (105 min). The run was complete after 114 min. An external calibration was carried out in MAXIS 3G—Bruker Daltonics 4.3 software to check for mass precision and data analysis. The tentative identification of the compounds was performed by comparing their exact mass (*m*/*z*) and MS^2^ spectra in negative mode to the database available in the literature and commercial standards (naringenin, pinocembrin, isoliquiritigenin, daidzein, formononetin andbiochanin A) from Sigma-Aldrich (St. Louis, MO, USA).

### 2.7. Data Analysis

STATISTICA 7.0 software was used to analyze the multivariate factorial design (ANOVA) and to determine the significance of the variables. The accuracy of the mathematical models was estimated by the coefficient of determination (R^2^) and the F-test (*p* < 0.05). All assays were carried out in triplicate, and the values were expressed as mean ± standard deviation.

## 3. Results and Discussion

### 3.1. Fitting the Models to Data

In this study, ANOVA was used to evaluate the accuracy of the RSM models (Table 2). The *p*-values (*p* < 0.05) observed for both response variables were considered significant, indicating that the developed models are appropriate to represent the relationship between the independent parameters and the response variables.

Based on the statistical analysis, the F-values observed (603.14 and 34.75 for TEAC and TPC, respectively) were significant and the model fitted well, as the *p*-value was lower than 0.05. The *p*-value is used to estimate whether F is large enough to indicate statistical significance, and values lower than 0.05 for this parameter indicate that the developed model is statistically significant [18]. Hence, the independent variables affected the TEAC and TPC. The *p*-values for Lack of Fit in both models were greater than 0.05 (0.3673 and 0.9251, TEAC and TPC respectively). This function is performed by comparing the variability of the residuals in the current model with the variability in the observations under repeated conditions of the factors [19]. The coefficient of determination (R^2^) estimates the proportion of variation in the response that can be attributed to the model rather than to random error [20]. In our study, the R^2^ values were 0.9991 and 0.9843 for TEAC and TPC, respectively, whereas the Adj-R^2^ values were 0.9974 and 0.9559 for TEAC and TPC. High Adj-R^2^ values are indicative of a high correlation between the observed and the predicted values [18]. Based on the estimated results, it is evident that the developed models have high adequacy and accuracy. 

The contribution of coefficients for the response variable is presented in Table 2. It can be observed that the linear ($X_{1}$, $X_{2}$ and $X_{3}$) and quadratic terms ($X_{2}^{2}$ and $X_{3}^{2}$) and the interactive effects ($X_{2}X_{3}$) affected the TEAC, while in TPC the highly significant terms were the linear ($X_{1}$), quadratic ($X_{1}^{2},\;X_{2}^{2}$ and $X_{3}^{2}$) and interactive ($X_{2}X_{3}$). The other coefficients were not significant (*p* > 0.05). Equations considering only the significant terms by RSM models were fitted to predict the responses, which are given below:

(2) $$Y_{TEAC}=13117.53+3.05X_{1}-60.23X_{2}-259.73X_{3}+0.31X_{2}^{2}\;+1.68X_{3}^{2}+0.41X_{2}X_{3}$$

(3) $$Y_{TPC}=135.01-4.11X_{1}-60.23X_{2}-3.26X_{1}^{2}-8.24X_{2}^{2}-4.70X_{3}^{2}\;-0.52X_{2}X_{3}$$

where, *Y* represents the predicting responses;and $X_{1}$, $X_{2}$ and $X_{3}$ represent time, temperature and % ethanol, respectively.

### 3.2. Response Surface Analysis

#### 3.2.1. The Effect of Solvent Concentration on TEAC and TPC

**Figure 1:**
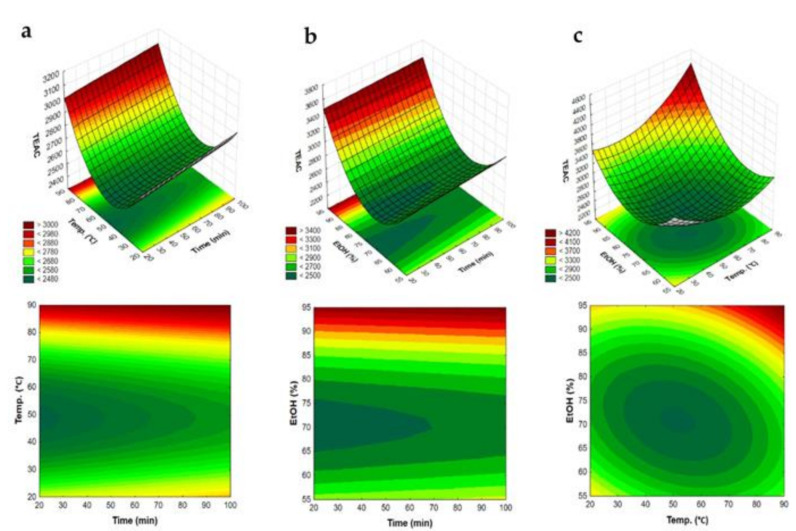
Response surface plot showing the combined effect of temperature (°C) (**a**), time (min) (**b**), and EtOH (%) (**c**) on the TEAC of Brazilian red propolis extracts.

**Figure 2:**
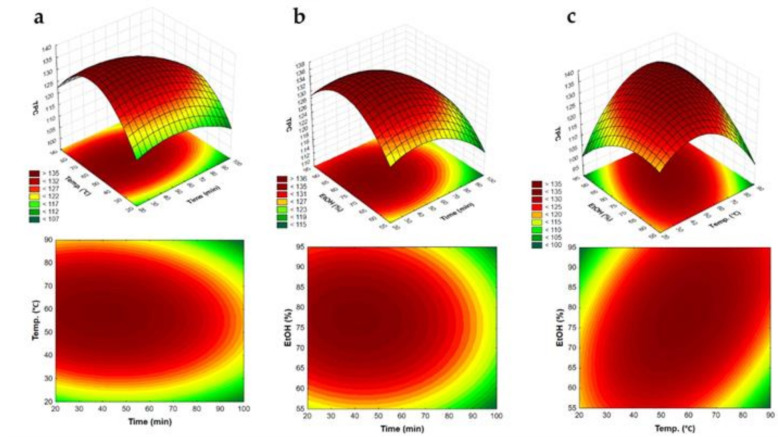
Response surface plot showing the combined effect of temperature (°C) (**a**), time (min) (**b**), and EtOH (%) (**c**) on the TPC of Brazilian red propolis extracts.

As shown in Figure 1 and Figure 2, solvent concentration significantly altered the content of bioactive compounds recovered from the BRP extract and its antioxidant activity. As the ethanol concentration increased, the efficiency of both phenolic extraction and antioxidant activity was greatly improved. TEAC values raised from 2700 to 3200 µmol TE/g as the ethanol concentration was increased from 75% to 90%. Similarly, TPC values raised from 123 to 135 mg GAE/g as the ethanol concentration was increased from 60% to 90%.

Our data suggest that the interaction between temperature and ethanol concentration was highly significant. Hence, the phenolic content and TEAC values can be optimized if these parameters are increased. Yet, no improvement in phenolic extraction and antioxidant activity was observed at the highest ethanol concentration (90%) and temperatures below 30 °C. A similar outcome was reported by Roselló-Soto et al. [21] during the optimization of Tiger Nuts byproducts. The authors showed that an increase in ethanol concentration significantly increased the extraction yield at temperatures above 40 °C. Oldoni et et al. [22] also reported that the optimized conditions for extraction of phenolic compounds with antioxidant activity from propolis were 80 °C and 70% ethanol. As proposed by Yang et al. [23], after modifying and penetrating through the cell wall, ethanol affects cell components and improves the chemical extraction, particularly of polyphenols.

Although it has not been extensively discussed in optimization studies, the extraction equipment plays a determining role in the efficiency of the optimization process. When experiments are carried out in non-sealed tubes, volatile solvents or nonpolar compounds may evaporate as rocking and sonification produce heat and increase the solvent temperature. Therefore, the use of an appropriate flask to perform the analysis is as important as the experimental design itself.

#### 3.2.2. The Effect of Temperature on TEAC and TPC

Temperature is one of the variables most frequently examined in natural product optimization studies due to its effects on the content and availability of bioactive compounds, mainly polyphenols. In our study, we found that as the temperature increased so did the TEAC of the extract. The best results were obtained at the highest tested temperature (80°C) 3471.75 µmol TE/g (Figure 1). This value is higher than those found by Andrade et al. [24] when testing the extraction of red, green, and brown propolis at 35 °C (2913.55 ± 95.26; 2214.96 ± 20.61 and 1868.45 ± 131.39 µmol TE/g, respectively).

Moreover, the results showed that when the temperature was escalated from 30 °C to 55 °C, the TPC increased from 122 to 128 mg GAE/g, but it decreased slightly when the temperature was further extended (Figure 2). Maran at al. [18] pointed out that higher temperatures enhance the efficiency of phenolic extraction by decreasing the viscosity and density of the extract. Thereby, higher temperatures enable the solvent to penetrate deeper into the sample matrix and have more contact with the surface area. However, if the temperature is excessively elevated, bioactive compounds may decompose or vaporize.

#### 3.2.3. The Effect of Extraction Time on TEAC and TPC

Figure 1 and Figure 2 show the results of different extraction times on TEAC and TPC outcomes. Although this variable was not strongly associated with TEAC values, a shorter extraction time (30 min) yielded better results. As for TPC, the optimal extraction time was also 30 min. When the time was extended from 60 to 90 min, the number of phenolic compounds recovered was drastically reduced. As stated in the literature, an extended extraction time increases the probability of oxidation, epimerization, and degradation of bioactive compounds [25]. Thus, a prolonged extraction procedure may not be appropriate for all types of natural products [21]. On the other hand, Oldoni et al. [22] reported an increase in the TPC of a propolis type produced in Southern Brazil when using 80% ethanol at 70 °C for 45 min. Yusof et al. [26] observed that the optimal conditions for extraction of phenolics from a Malaysian propolis were 80% ethanol at 60 °C for 25 min. Importantly, we note that the optimal extraction time reported by these authors was shorter than that found in our study, and that it was not possible to predict the extraction efficiency after 60 min.

#### 3.2.4. Optimization and Validation of RSM Models

The optimization of the independent parameters–time (min), ethanol (%) and temperature (°C)—was carried out based on the desirability coefficient (0.8780) to obtain the highest TEAC and TPC (See Supplementary Materials Figure S1). The TEAC and TPC values predicted by the model under optimal conditions (90% ethanol, 80°C, 30 min) were 3550.8 ± 70 µmol TE/g and 132 ± 6.48 mg GAE/g, respectively. The RSM model was validated by comparing the experimental data (*n* = 3) with the predicted values. The actual TEAC and TPC values obtained under optimal conditions were 3471.76 ± 53.86 µmol TE/g and 129.00 ± 2.16 mg GAE/g, respectively. These findings are similar to the predicted data, indicating that the method is suitable to determine the optimal conditions for extraction of phenolic compounds with antioxidant activity from BRP samples.

### 3.3. Antioxidant Activity of the Optimized BRP Extract

The antioxidant activity of the optimized BRP extract was evaluated in vitro by single electron transfer and hydrogen atom transfer assays. The FRAP method is based on the reduction of Fe^3+^ into Fe^2+^ by antioxidant compounds in the presence of 2,4,6-tris-(2-pyridyl)-s-triazine (TPTZ), forming a colored complex with Fe^2+^ at 593 nm [27]. The FRAP value obtained for the optimized BRP extract was 1472.86 ± 72.37 µmol Fe^2+^/g of dry extract. These values were higher than those reported by Calegari et al. [27], who determined the antioxidant activity of 30 propolis samples collected in the states of Paraná and Santa Catarina, Brazil. The authors used Fourier transform near-infrared (FTNIR) spectroscopy and obtained FRAP values ranging from 61.9 to 1770 µmol Fe^2+^/g of dry weight. Andrade et al. [24] reported a FRAP value of 633.18 ± 40.20 µmol TE/g of dry weight for the BRP extract, while Oldoni et al. [22] found 259.30 ± 9.50 µmol Fe^2+^/g of dry weight for optimized propolis samples from the state of Paraná, Brazil. When evaluating Croatian propolis from five locations in Adriatic Sea islands, Sveĉnjak et al. [28] observed reducing FRAP activity from 0.1 to 0.8 mmol Fe^2+^/g of dry weight.

The oxygen radical absorbance capacity (ORAC) assay measures antioxidant inhibition of the peroxyl radical via hydrogen atom transfer reactions. This method is suitable to detect both hydrophilic and hydrophobic antioxidants [29]. For that reason, it is commonly used to determine the antioxidant capacity of different types of natural products, including propolis. In our study, the ORAC value of the BRP extract was 4339.61 ± 114.65 µmol TE/gof dry extract. El-Guendouz et al. [30] examined 24 different samples of Moroccan propolis and found ORAC values ranging from 630.39 ± 33.79 to 1723.28 ± 33.79 µmol TE/g of dry weight. Using 95% ethanol for extraction, Sun et al. [31] reported that Beijing propolis extract had an ORAC value of 1433.72 ± 120 µmol TE/g of dry weight. Finally, a high correlation between the phenolic composition and antioxidant activity of the optimized extract was observed in our study (ABTS, r^2^ = 0.9925; FRAP = −0.7321 and ORAC = 0.8152). 

### 3.4. Characterization of Phenolic Compounds in the Optimized BRPExtract by LC-ESI-QTOF-MS/MSAnalysis

The qualitative analysis of the BRP extract composition was carried by LC-ESI-QTOF-MS/MS. The compounds were tentatively identified by comparing their *m*/*z* values and MS^2^ spectra in negative mode to the literature findings and corresponding standards.

As shown in Table 3, LC-MS/MS analysis revealed the presence of 32 phenolic compounds in the optimized BRP extract, including flavones, flavanones, flavanonols, chalcones, isoflavonoids, quinone, coumarin, and their derivatives.

#### 3.4.1. Flavonoids

Flavonoids are the main class of phenolic compounds in several natural products, including fruits, vegetables, roots, stems and flowers [32]. Multiple studies have revealed the beneficial effects of flavonoids extracted from propolis against human diseases [33]. In our study, a total of 28 flavonoids were identified in the BRP extract, which corresponded to the main chemical group present in the sample.

#### 3.4.2. Flavones

Among the flavonoids detected in the BRP extract, six were flavones. Chrysin (compound **1** with [M–H]^−^ at *m/z* 253.0510) yielded a fragment at *m*/*z* 253.0507 [34]. Tricin (compound **2** with [M–H]^−^ at *m*/*z* 329.0667) was tentatively identified based on product ions at *m*/*z* 329.0667, 299.0218 [M–H–2CH3]^−^, 271.0263 [M–H–C_2_H_2_O_2_]^−^, and 243.0289 [M–H–C_4_H_6_O_2_]^−^. Genkwanin (compound **3** with [M–H]^−^ at *m*/*z* 283.0624) yielded the predominant *m*/*z* 268 fragment due to the loss of CH_2_ from the *m*/*z* 283 fragment, resulting in a stable fragment structure [35]. Hispidulin (compound **4**) was tentatively identified based on the [M–H]^₋^ ion at *m*/*z* 299.0565, with fragment ions at *m*/*z* 284.0331 [M–CH_3_], 227.0354 [M–CO_2_–CO]; 255.0301 [M–H–CO_2_] and 212.0483 [M–H–CO_2_–CO–CH_3_] [34]. Compound **5** was tentatively characterized as 8-Hydroxy-5-methoxyflavanon (*m*/*z* 269.0831) based on the *m*/*z* 254.0589 fragment. Finally, the characteristic [M–H]^−^ ion at *m*/*z* 283.0617 and a major fragmentation at *m*/*z* 268.0382 were suggestive of acacetin (compound **6**) [36].

#### 3.4.3. Flavanones

Retro–Diels–Alder (RDA) is the pathway fragmentation commonly used by flavanones. Fragment ions resulting from RDA fragments are more abundant than the loss of other radical ions, such as CH_3_, CO, OH, or H_2_O [37]. Liquiritigenin (compound **7**) was detected with [M–H]^−^ at *m*/*z* 255.0667. Its identity was confirmed by comparing with data from a previous study, in which *Dalbergia odorifera* was characterized by LC-MS/MS and based on the spectrum of product ions at *m*/*z* 119.0495 ([M–H–C_8_H_8_O_2_]^−^) and *m*/*z* 135.0083, corresponding to breaks of [1,3A–H]^−^ and [1,3B–H]^−^ fragmen [38]. Four flavanones and derivates (compounds **8**, **9**, **10** and **11**) were tentatively identified in the optimized BRP extract as naringenin, pinocembrin, 5,6-Dihydroxy-3′,4′-dimethoxyflavanon and 6-Hydroxyflavanone, according to the precursor ions [M–H]^−^ at *m*/*z* 271.0619, 255.0668, 315.0882 and 239.0722, respectively. The identification of naringenin was confirmed by a product ion at *m*/*z* 119.0487 [39]. Pinocembrin was identified by comparing our findings with those of a previous report, where this compound was found in leaf extracts of *Alpinia zerumbe*, yielding the *m*/*z* 255.0678 fragment [40]. Pinocembrin is an important marker in BRP, because it is also found in *D. ecastaphyllum* [9]. The compound 5,6-Dihydroxy-3′,4′-dimethoxyflavanon (compound **10** with [M–H]^−^ at *m*/*z* 315.0882), which was found for the first time in BRP, was tentatively identified based on a product ion at *m*/*z* 315.0881. Lastly, 6-Hydroxyflavanone displayed a product ion at *m*/*z* 239.0732 in the MS^2^ spectra [37].

#### 3.4.4. Chalcones

Isoliquiritigenin (compound **12** with [M ₋ H]¯ at *m*/*z* 255.0676) was previously described in the literature and tentatively identified herein based on product ions at *m*/*z* 119.0496 and 135.0082 [41]. The compound 2′,4′-Dihydroxychalcone (compound **13**), detected with [M–H]^−^ at *m*/*z* 239.0723, was identified based on fragment ions at *m*/*z* 239.0709, 197.0609 and 135.0085 [42] This compound was previously reported as an efficient antiviral targeting HlyU in *Vibrio vulnificus* [43]. Compound **14** was assigned as 7-hydroxyflavanone (*m*/*z* 239.0719), yielding the *m*/*z* 197.0610 fragment [37]. Compound **15** was detected with [M–H]^−^ at *m*/*z* 271.0990 and was tentatively identified as 2′,6′-dihydroxy-4′-methoxydihydrochalcone based on fragment ions at *m*/*z* 254.0590, 135.0444 and 109.0287. Compound **16**, with [M–H]^−^ at *m*/*z* 271.0990, was identified as 2′-Hydroxy-4′-methoxychalcone (C1_6_H1_3_O_3_^−^), yielding fragment ions at *m*/*z* 237.0552, 255.0665 and 253.0872. This compound was previously found in orange-yellow resin from *Zuccagnia punctate* [19].

#### 3.4.5. Isoflavonoids

The isoflavones aglycones daidzein (compound **17**, *m*/*z* 253.0511), formononetin (compound **22**, *m*/*z* 267.0666) and biochanin A (compound **25**, *m*/*z* 283.0619) were detected in the BRP extract. Daidzein yielded a product ion at *m*/*z* 253.0513 [M–C_6_H_10_O_5_]^−^ as result of a loss of glucoside and another product ion at *m*/*z* 135.0089 [M–H–C_8_H_6_O]^−^ [44]. The main fragment ions in the MS^2^ spectra of formononetin corresponded to successive losses of CH^3^, CHO, and CO [39]. Its MS^2^ spectra showed fragment ions at *m*/*z* 252.0431 [M–H–CH_3_]^−^, 223.0404 (C_14_H_7_O_3_) [M–H–CH_3_–CHO]^−^ and 195.0456 [M–H–CH_3_–CHO–CO]^−^. A fragment ion at *m*/*z* 268.0389 [M–H–CH_3_]^−^, which was produced due to the loss of a CH_3_ group, and ions at *m*/*z* 239.0354 [M–CO_2_] and 211.0393 [M–CO_2_–CO], were suggestive of Biochanin A fragmentation [34]. Compound **18**, with [M−H]^−^ at *m*/*z* 283.0617 (C_16_H_11_O_5_), showed typical product ions at *m*/*z* 268.0353 (C_15_H_8_O_5_), 211.0422, 224.0506 and 239.0313. Therefore, it was tentatively classified as calycosin [45]. Dihydrobiochanin A (compound **19** with [M–H]^−^ at *m*/*z* 285.0776) and vestitone (compound **20** with [M–H]^−^ at *m*/*z* 285.0776) were characterized based on fragment ions at *m*/*z* 270.0541 and 270.0535, respectively [46,47]. Vestitol (compound **21**), with [M–H]^−^ at *m*/*z* 285.0776 (C_16_H_15_O_4_), displayed product ions at *m*/*z* 135.0450 (C_8_H_7_O_2_), 109.0282 (C_6_H_5_O_2_) and 149.0604 [48]. Even though compound **22** ([M–H]^−^ at *m*/*z* 271.0990) showed a similar fragment to vestitol, it was tentatively identified as neovestitol based on the main fragment ions at *m*/*z* 135.0360,197.0482 and 212.0707 [5]. Vestitol and neovestitol have been previously isolated from BRP and were reported to have strong biological properties [5,9]. Compound **24** ([M–H]^−^ at *m*/*z* 255.0673) was tentatively identified as dimethyl medicarpin based on fragment ions at *m*/*z* 255.0668, 151.0032, 107.0118 and 213.0532. Compound **25** was characterized as medicarpin according to the precursor ion at *m*/*z* 269.0827. In its MS^2^ spectra, the following typical product ions were detected: 254.0594([M–H–CH3^•^]^−^), 225.0540, 105.0191, 121.0300 (C_7_H_5_O_2_, ^3,5^A^−^)and 133.0287 [49]. Compound **27** (with [M–H]^−^ at *m*/*z* 283.0619) and compound **28** (with [M–H]^−^ at *m*/*z* 283.0988) were tentatively identified as 5,1′-Dihydroxy-7-methoxyisoflavone and 3,9-Dimethoxypterocarpan, respectively. In their MS^2^ spectra, 5,1′-Dihydroxy-7-methoxyisoflavone displayed fragment ions at *m*/*z* 268.0383 [M–H–CH_3_], 211.0422 [M–CO_2_–CO] and 223.0402 [M–H–CO–H_2_O] [34].

#### 3.4.6. Flavonols, Neoflavonoids, Coumarins, and Polyprenylated Benzophenone Derivates

Dalbergin (compound **30** with [M–H]^−^ at *m*/*z* 267.0667) yielded product ions at *m*/*z* 252.0465 and 224.0503, corresponding to the loss of a CO_2_ and further loss of H_2_O from the precursor ion [47]. Compound **29** (with [M–H]^−^ at *m*/*z* 285.0743) was tentatively identified as 7-Hydroxy-6-methoxydihydroflavonol based on fragment ions at *m*/*z* 270.0534. Guttiferone E (Compound **31**) displayed deprotonated molecular ion at *m*/*z* 601.3571. Its MS^2^ spectra showed fragment ions at *m*/*z* 109.0291 [C_6_O_2_H_5_]^−^, 108.0214, 202.9997, 177.0198 [M–H–C_10_H_16_O]^−^, 335.1285 [50]. Lastly, compound **32** (with [M–H]^−^ at *m*/*z* 601.3670) was tentatively identified as oblongifolin B, yielding a fragment ion at *m*/*z* 109.0292.

In addition to comparing the tentative compounds with the literature, we further compared the data against the electronic database available from metadata-centric approaches, such as the Mass Bank of North America (MoNA) and the Mass Bank. The LC-QTOF-ESI-MS/MS analysis in negative mode enabled the identification for the first time in BRP of the following phenolic compounds: Flavones (tricin, genkwanin, hispidulin and 8-Hydroxy-5-methoxyflavanone), flavanones (5,6-Dihydroxy-3′,4′-dimethoxyflavanone and 6-Hydroxyflavanone), chalcones (2′,4′-Dihydroxychalcone and 2′,6′-dihydroxy-4′-methoxydihydrochalcone), isoflavonoids (dihydrobiochanin A, demethyl medicarpin, 5,4′-Dihydroxy-7-methoxyisoflavone and 3,9-Dimethoxypterocarpan) and flavanols (7-Hydroxy-6-methoxydihydroflavonol).

## 4. Conclusions

The optimization of conditions for extraction of antioxidant compounds from BRP extract was successfully performed using the Response Surface Methodology. The optimal extraction conditions for stronger antioxidant activity and higher phenolic content were 90% ethanol at 80 °C for 30 min. Under optimized conditions, BRP extract showed a TPC of 129.00 mg GAE/g. When examined for its antioxidant activity, the TEAC, FRAP and ORAC assays revealed that the optimized extract had 3471.76 µmol TE/g, 1472.86 µmol Fe^2+^/g and 4339.61 µmol TE/gof dry extract, respectively. Thirty-two phenolic compounds were tentatively identified by LC-QTOF-ESI-MS/MS, of which thirteen were found for the first time in BRP. Our study may guide further research in the field, since this is the first study that reported the optimization of the extraction of phenolic compounds from BRP samples. Collectively, our results highlight the importance of BRP as a source of a wide variety of phenolic compounds with significant antioxidant properties.

## Figures and Tables

**Table 1:** Box–Behnken Design for the extraction of antioxidants compounds from Brazilian red propolis extracts.

Run	Independent Variables					Dependent Variables		
	Coded Values			Real Values			TEAC (µmol TE/g)	TPC (mg GAE/g)
	Time (min)	Temp. °C	EtOH (%)	Time (min)	Temp. °C	EtOH (%)		
1	−1	−1	0	30	30	75	2560.36	125.76
2	1	−1	0	90	30	75	2386.42	132.80
3	−1	1	0	30	80	75	2804.74	136.17
4	1	1	0	90	80	75	2918.30	118.82
5	−1	0	−1	30	55	60	2370.61	120.30
6	1	0	−1	90	55	60	2719.93	116.09
7	−1	0	1	30	55	90	3106.62	127.64
8	1	0	1	90	55	90	3156.93	123.38
9	0	−1	−1	60	30	60	3169.87	118.82
10	0	1	−1	60	80	60	2827.74	116.53
11	0	−1	1	60	30	90	3200.06	109.49
12	0	1	1	60	80	90	3471.75	126.25
13	0	0	0	60	55	75	2544.55	133.00
14	0	0	0	60	55	75	2635.12	137.06
15	0	0	0	60	55	75	2622.18	134.98

Temp. = Temperature; EtOH = Ethanol; TEAC= Trolox equivalent antioxidant capacity; TPC= Total phenolic compounds.

**Table 2:** The effects of different parameters on Trolox equivalent antioxidant capacity (TEAC) and total phenolic content (TPC) outcomes during optimization of Brazilian red propolis ethanolic extracts.

Term	SS	df	MS	F-Value	*p*-Value	Remarks
TEAC						
Model	1,247,423.00	9	138,602.55	603.14	<0.0001	significant
$X_{1}$	8019.54	1	8019.54	34.89	0.0183	
$X_{2}$	74,849.67	1	74,849.67	325.63	0.0020	
$X_{3}$	425,485	1	425,484.7	1851.06	0.0003	
$X_{1}^{2}$	83.58	1	83.58	0.3636	0.5347	
$X_{2}^{2}$	144,174	1	144,173.8	627.22	0.0010	
$X_{3}^{2}$	530,460	1	530,460.1	2307.80	0.0002	
$X_{1}X_{2}$	1153.35	1	1153.35	5.02	0.1092	
$X_{1}X_{3}$	1.87	1	1.87	0.0081	0.9254	
$X_{2}X_{3}$	94,179.02	1	94,179.02	409.73	0.0016	
Residual	1149.29	5	229.86			
Lack of Fit	846.99	3	282.33	1.87	0.3673	non-significant
Pure error	302.30	2	151.15			
Total	1,248,572	14				
R^2^	0.9991					
Adj-R^2^	0.9974					
TPC						
Model	627.04	9	69.67	34.75	0.0006	significant
$X_{1}$	134.88	1	134.88	67.27	0.0004	
$X_{2}$	10.38	1	10.38	5.18	0.0719	
$X_{3}$	9.45	1	9.45	4.71	0.0820	
$X_{1}^{2}$	39.29	1	39.29	19.60	0.0068	
$X_{2}^{2}$	250.65	1	250.65	125.01	<0.0001	
$X_{3}^{2}$	81.65	1	81.65	40.72	0.0014	
$X_{1}X_{2}$	5.97	1	5.97	2.98	0.1449	
$X_{1}X_{3}$	1.10	1	1.10	0.5473	0.4927	
$X_{2}X_{3}$	132.72	1	132.72	66.20	0.0005	
Residual	10.02	5	2.00			
Lack of Fit	1.78	3	0.5938	0.1441	0.9251	non-significant
Pure error	8.24	2	4.12			
Total	637.07	14				
R^2^	0.9843					
Adj-R^2^	0.9559					

X_1_: Time (min), X_2_: Temperature (°C), X_3_: EtOH (%), SS: Sum of squares, DF: Degree of freedom, MS: Mean square, R^2^: Quadratic correlation coefficient; Adj-R^2^: Adjusted quadratic correlation coefficient. TEAC: Trolox equivalent antioxidant capacity; TPC: Total phenolic content.

**Table 3:** LC-ESI-QTOF-MS/MS analysis of phenolic compounds present in Brazilian red propolis.

Compound	Putative Compound Name	RT (min)	ProposedFormula	[M–H]^−^(*m*/*z*)	MS/MS Fragments (*m*/*z*)
Flavones					
1	Chrysin	28.6	C_15_H_10_O_4_	253.0510	**253.0507**; 119.0483; 195.0438; 224.0481; 209.0614
2	Tricin	34.2	C_17_H_14_O_7_	329.0677	**329.0667**; 299.0218; 271.0263; 243.0289
3	Genkwanin	36.2	C_16_H_12_O_5_	283.0624	**268.0360**; 283.0583; 269.0397
4	Hispidulin	37.5	C_16_H_12_O_6_	299.0565	**284.0331**; 227.0354; 255.0301; 212.0483
5	8-Hydroxy-5-methoxyflavanone	44.2	C_16_H_14_O_4_	269.0831	**254.0589**; 252.0437; 195.0451; 210.0685
6	Acacetin	54.2	C_16_H_12_O_5_	283.0617	**268.0382**; 211.0408; 269.042
Flavanones					
7	Liquiritigenin	25.1	C_15_H_12_O_4_	255.0667	**119.0495**; 135.0083; 255.0656; 120.0526
8	Naringenin *	32.6	C_15_H_12_O_5_	271.0619	**119.0487**; 151.0029; 254.0596; 271.0609; 165.0207
9	Pinocembrin *	48.6	C_15_H_12_O_4_	255.0668	**255.0678**; 240.0426; 151.0034; 133.0285; 213.0540;
10	5,6-Dihydroxy-3′,4′-dimethoxyflavanone	48.7	C_17_H_16_O_6_	315.0882	**315.0881**;151.0037; 235.0636; 255.1042; 121.0292;
11	6-Hydroxyflavanone	57.3	C_15_H_12_O_3_	239.0722	**239.0732**; 135.0091; 197.0643
Chalcones					
12	Isoliquiritigenin *	41.1	C_15_H_12_O_4_	255.0676	**119.0496**; 135.0082; 120.0531; 151.0384; 255.0665
13	2′,4′-Dihydroxychalcone	41.9	C_15_H_12_O_3_	239.0723	**239.0709**; 197.0609; 135.0085; 198.0667
14	7-hydroxyflavanone	42.2	C_15_H_12_O_3_	239.0719	**197.0610**; 135.0085; 239.0732; 198.0643
15	2′,6′-dihydroxy-4′-methoxydihydrochalcone	45.2	C_16_H_16_O_4_	271.0990	**254.0590**; 135.0444; 109.0287;
16	2′-Hydroxy-4′-methoxychalcone	49.9	C_16_H_14_O_3_	253.0879	**237.0552**; 255.0665; 253.0872; 136.0169; 161.0239
Isoflavonoids					
17	Daidzein *	28.7	C_15_H_10_O_4_	253.0511	**253.0513**; 208.0523; 119.0488; 135.0089
18	Calycosin	31.9	C_16_H_12_O_5_	283.0617	**268.0353**; 211.0422; 224.0506; 239.0313; 267.0665
19	Dihydrobiochanin A	34.1	C_16_H_14_O_5_	285.0776	**270.0541**; 109.0289; 161.0242; 285.0767
20	Vestitone	34.5	C_16_H_14_O_5_	285.0776	**270.0535**; 161.0240; 109.0286; 271.0607
21	Vestitol	41.4	C_16_H_16_O_4_	271.0987	**135.0450**; 109.0282; 149.0604; 147.0452; 271.0986; 256.0747
22	Neovestitol	41.8	C_16_H_16_O_4_	271.0990	**135.0360**; 109.0217;256.0555; 197.0482; 212.0707
23	Formononetin *	43.9	C_16_H_12_O_4_	267.0666	**252.0431**; 254.0594; 223.0404; 195.0456; 253.0483
24	Demethyl medicarpin	45.2	C_15_H_12_O_4_	255.0673	**255.0668**; 105.0189; 151.0032; 107.0118; 213.0532
25	Medicarpin	48.6	C_16_H_14_O_4_	269.0827	**254.0594**; 225.0540; 105.0191; 121.0300; 133.0287
26	Biochanin A *	52.0	C_16_H_12_O_5_	283.0619	**268.0389**; 239.0354; 211.0393; 132.0202;195.4450
27	5,4′-Dihydroxy-7-methoxyisoflavone	53.1	C_16_H_12_O_5_	283.0619	**268.0383**; 211.0422; 223.0402; 224.0506;
28	3,9-Dimethoxypterocarpan	63.0	C_17_H_16_O_4_	283.0988	**253.0515**; 225.0564; 268.0754; 183.0456; 254.0554
Flavonols					
29	7-Hydroxy-6-methoxydihydroflavonol	30.9	C_16_H_14_O_5_	285.0743	**270.0534**; 268.0383; 78.9984; 123.0078
Neoflavonoids					
30	Dalbergin	38.3	C_16_H_12_O_4_	267.0667	**252.0465**; 224.0503; 195.0451; 267.0650; 204.9615
Polyprenylated benzophenones					
31	Guttiferone E/Xanthochymol	92.2	C_38_H_50_O_6_	601.3567	**109.0291**; 108.0214; 202.9997; 177.0198; 335.1285
32	Oblongifolin B	93.8	C_38_H_50_O_6_	601.3569	**109.0292**; 108.0216; 176.0146; 307.1362

Bold values indicate the main fragments; RT = retention time; [M−H]^−^ (negative ionization mode) experimental mass of compound. * As compared to an authentic standard.

## Data Availability

Data is contained within the article and Supplementary Materials.

## Supplementary Materials

The following are available online at https://www.mdpi.com/2076-3921/10/2/297/s1, Figure S1 Coefficient of desirability for optimization of TEAC and TPC in Brazilian red propolis extracts as a function of time (min), temperature (°C) and EtOH concentration (%).
