# Supplementary material for: Active Antioxidant Phenolics from Brazilian Red Propolis: An Optimization Study for Their Recovery and Identification by LC-ESI-QTOF-MS/MS
Source: Antioxidants (Basel). 2021 Feb 16;10(2):297. doi: 10.3390/antiox10020297 (PMC7919790; doi:10.3390/antiox10020297)
Supplement: Supplementary file 1 [file antioxidants-10-00297-s001.pdf]

Supplementary Material

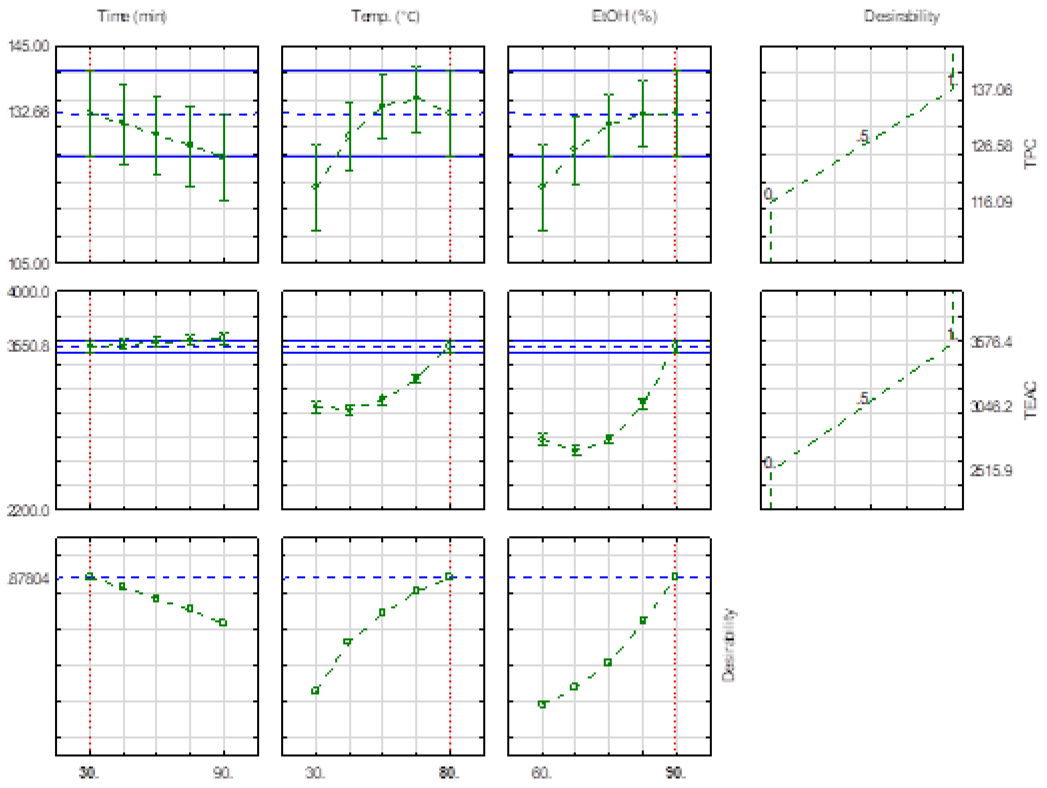

Figure S1. Response surface plot showing the combined effect of temperature (°C) (**a**), time (min) (**b**), and EtOH (%) (**c**) on the TEAC of Brazilian Red Propolis extracts.
